# A neotype designation for the bone-skipper *Centrophlebomyia anthropophaga* (Diptera, Piophilidae, Thyreophorina), with a review of the Palaearctic species of *Centrophlebomyia*

**DOI:** 10.3897/zookeys.310.4914

**Published:** 2013-06-17

**Authors:** Maurizio Mei, Daniel Whitmore, Giuseppe Lo Giudice, Pierfilippo Cerretti

**Affiliations:** 1 Dipartimento di Biologia e Biotecnologie “Charles Darwin”, Università di Roma “La Sapienza”, Piazzale A. Moro 5, I 00185 Rome, Italy; 2 Department of Life Sciences, Natural History Museum, Cromwell Road, London, SW7 5BD, UK; 3 Centro Nazionale Biodiversità Forestale (CNBFVR) – Corpo Forestale dello Stato, via Carlo Ederle 16/a, 37100 Verona, Italy

**Keywords:** Neotype designation, new synonymy, *Protothyreophora*, preimaginal instars, life history, systematics

## Abstract

The European bone-skippers (Diptera: Piophilidae: Thyreophorina), long considered extinct, have recently been the object of much interest by dipterists after their unexpected rediscovery. Considerable faunistic work has been done on these flies in recent years. However, some nomenclatural and taxonomic issues still require attention. A neotype is designated for *Thyreophora anthropophaga* Robineau-Desvoidy, 1830 (now in the genus *Centrophlebomyia* Hendel, 1903) to fix the identity of this nominal species. *Centrophlebomyia anthropophaga* is recognized as a valid species. It is described and illustrated in detail, and information on its preimaginal instars is provided for the first time. Four Palaearctic species of *Centrophlebomyia* are recognized and reviewed and a key is provided for their identification. *Centrophlebomyia orientalis* Hendel, 1907 from northern India, is removed from synonymy with *Centrophlebomyia anthropophaga* and recognized as a valid species of *Centrophlebomyia*, **stat. r.** The nominal genus *Protothyreophora* Ozerov, 1984 is considered a junior synonym of *Centrophlebomyia*, **syn. n.**

## Introduction

It is undeniable that some organisms are able to provoke great curiosity, which may last through generations of students. There are several, often interrelated reasons for this: rarity for example, true or apparent, but also a certain aesthetic appeal or unusual biology. All of these factors have contributed to the long-term popularity of thyreophorine Piophilidae, particularly that of the European species. These unusual-looking flies, commonly known as bone-skippers, appear to be associated as necrophages with large vertebrate carrion, including human corpses, and have always been considered rare, almost legendary (*cf.* Pape 2009). The three European thyreophorines, *Centrophlebomyia furcata* (Fabricius, 1794), *Centrophlebomyia anthropophaga* (Robineau-Desvoidy, 1830) and *Thyreophora cynophila* (Panzer, 1798), were even considered extinct for over a century. All three were rediscovered in the last 30 years, during which almost every record of a thyreophorine was published (Freidberg 1981; Michelsen 1983; Contini and Rivosecchi 1993; Gòmez-Gòmez et al. 2008; Martín-Vega and Baz 2011; Martín-Vega et al. 2010; Carles-Tolrá 2011; Carles-Tolrá et al. 2010, 2011, 2012; Zaldivar Ezquerro et al. 2011), sometimes with a certain media emphasis (Appendix 1). Despite the aura of exceptionality surrounding these flies, and the excitement their rediscovery has recently raised among dipterists, there is still some confusion concerning their taxonomy and nomenclature.

The present work was triggered by the finding of several specimens of a species of *Centrophlebomyia* Hendel, 1903 in central Italy (Monte Velino, Central Apennines), during a study on the necrophilous insect fauna associated with carrion (Lo Giudice 2007). These specimens are conspecific with those recorded 25 years ago from Sardinia as *Centrophlebomyia anthropophaga* (Contini and Rivosecchi 1993; Martín-Vega et al. 2010). However, their identification turned out to be trickier than expected and had to rely on the study of all known Palaearctic thyreophorines, including genus *Protothyreophora* Ozerov, 1984. This allowed us to review the taxonomy and nomenclature of the Palaearctic Thyreophorina, modifying the arrangement proposed for this group by Martín-Vega et al. (2010), and designating a neotype for *Centrophlebomyia anthropophaga*. We also provide the first ever information on preimaginal instars of *Centrophlebomyia anthropophaga* and an updated key to all Palaearctic species of the group.

## Material and methods

### Specimens

Male terminalia, pinned specimens and larvae were examined, uncoated, with a Hitachi TM1000 environmental scanning electron microscope (ESEM). Male terminalia were also slide mounted. Line drawings were made using a drawing tube. Figure 38 was prepared from composites of images captured using a DS-L1 digital camera (Nikon, Tokyo) mounted on a MZ 12.5 stereoscopic microscope (Leica, Wetzlar, Germany) and processed with Helicon Focus Pro software (Kharkov, Ukraine).

Male terminalia were dissected following the method described by Cerretti and Pape (2012) and, after examination, were preserved in glycerine in a plastic microvial pinned beneath the specimen.

The material examined is deposited in the following collections (acronyms as used in the text):

**MZUR** Museum of Zoology, Sapienza Università di Roma, Italy;

**NHMW** Naturhistorische Museum, Wien, Austria;

**TAU** Department of Zoology, Tel Aviv University, Tel Aviv, Israel;

**ZMUC** Natural History Museum of Denmark, Zoological Museum, University of Copenhagen, Denmark.

Label data of type specimens are given verbatim using the following symbols:

/ end of a line and beginning of the next;

// end of a label and beginning of the next (from top to bottom on the same pin).

### Terminology

Morphological terminology essentially follows Merz and Haenni (2000) except for the antenna, for which we follow Stuckenberg (1999). Measurements and ratios of the head follow Cerretti (2010).

## Results

### Taxonomy

#### Genus Centrophlebomyia

Hendel, 1903

http://species-id.net/wiki/Centrophlebomyia

Figs 1 −38

Centrophlebomyia Hendel 1903: 216. [original description] – type species: *Musca furcata* Fabricius, 1794: 343, by original designation.
Thyreolepida Sack 1939: 4. [original description] – type species: *Thyreolepida cinerea* Sack, 1939:4, by original designation.
Protothyreophora Ozerov 1984a: 465. [original description] – type species: *Protothyreophora grunini* Ozerov 1984a, by original designation; **syn. n.** (see below).

##### References.

Hendel 1903; McAlpine 1977; Ozerov 1984a; Ozerov 1984b; Ozerov 2000.

##### Recognition.

Brownish, scathophagid-like flies, body length 4−8 mm. Body densely to moderately microtomentose and covered with long, fine setulae, especially in males. Frons with one or two upper reclinate orbital setae. Ocellar seta, medial and lateral vertical setae, and postvertical seta long and robust. Two to ten frontal setae usually arranged more or less regularly around lunula. Face with a strong, flattened median carina, antennal grooves deep. Parafacial with a patch of microtomentum at mid length (Fig. 15). Compound eye almost round in lateral view. Two pairs of strong vibrissae present, subequal in length and strength. Thoracic chaetotaxy as follows: 0−2 postpronotal setae (postpronotal setae usually absent in male of *Centrophlebomyia furcata*); 1 + 1 intra-alar, 1−2 + 3 strong dorsocentral setae, 1 postalar, 2 notopleural setae, 0−1 prescutellar acrostichal setae. Scutellum long, dorsally flattened, much more developed in male than in female, with two pairs of setae, apical pair very long and strong, almost spiniform in larger males. Dorsal surface of scutellum bare. Development of scutellum in male related to body size. Propleural seta strong. Katepisternum densely setulose, with one strong katepisternal seta at upper posterior margin. Anepisternum with a row of setae along posterior margin, one of them strong. Thorax (except scutellum) very finely setulose throughout, besides the strong setae.

Wing membrane hyaline. Costa more or less spinose (i.e., with a regular row of stronger setulae interspersed with the general costal setulae), spine-like setae stronger in male than in female. Anal vein fading out well before wing margin. Legs thickly setulose in both sexes (almost woolly in male). Fore femur with 5–6 weak posteroventral setae near apex, scarcely differentiated in male. Hind femur with 2–3 anteroventral setae near apex. Mid tibia with five apical setae on ventral side: middle and lateral ones strongest. Hind tibia with one short, curved apical seta on posteroventral surface. Tarsi unmodified.

##### Preimaginal instars.

Described by Freidberg (1981) and below.

##### Distribution.

Europe, North Africa, Middle East, Russian Far East, northern India (Kashmir and Darjeeling).

##### Remarks.

The generic diagnosis incorporates the characters given by McAlpine (1977) based only on the type species *Centrophlebomyia furcata*.

**Figures 1−10.**
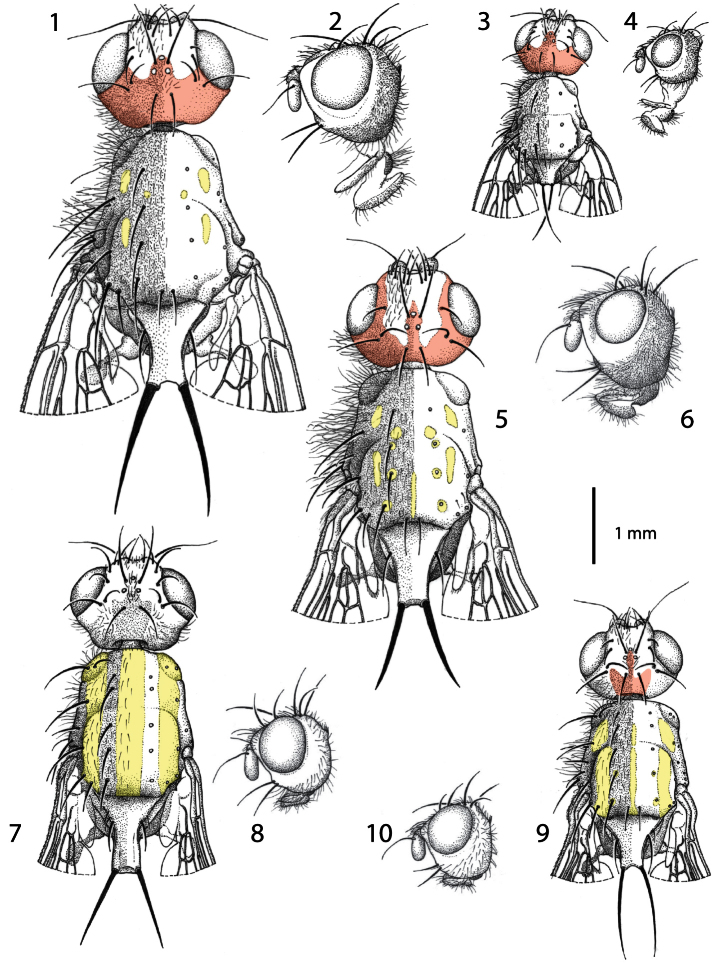
Males of *Centrophlebomyia* spp. **1**, **3**, **5**, **7**, **9** head and thorax in dorsal view **2**, **4**, **6**, **8**, **10** head in lateral view **1−4** *Centrophlebomyia anthropophaga* (Italy) **5−6** *Centrophlebomyia furcata* (Italy) **7−8** *Centrophlebomyia grunini* (Russian Far East) **9−10** *Centrophlebomyia orientalis* (India). In red the microtomentum pattern of head; in yellow the shiny, non microtomentose, pattern of thorax.

**Figures 11−15.**
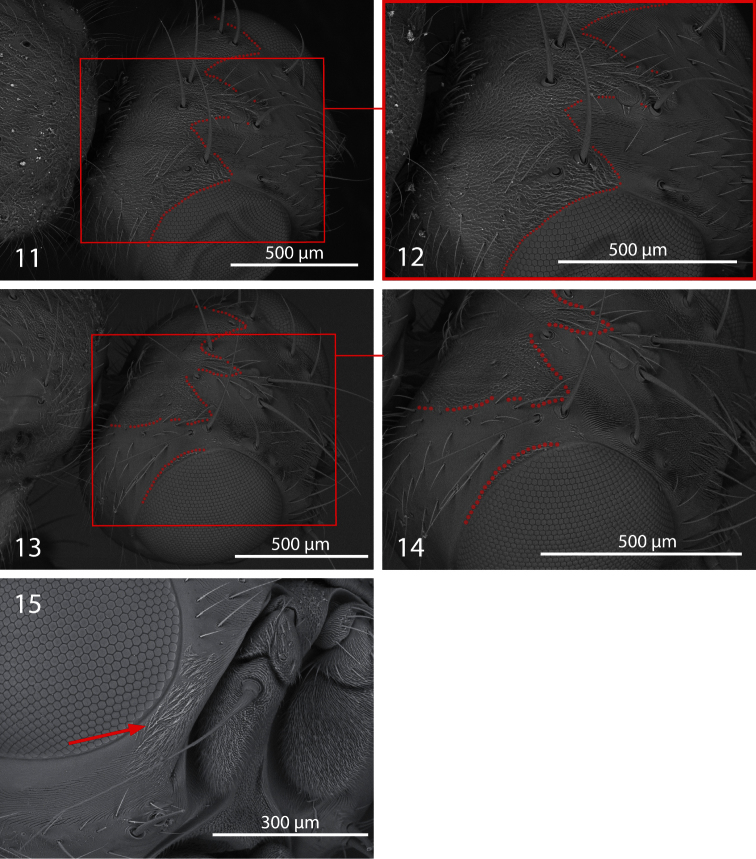
*Centrophlebomyia* spp. **11−14** head in dorsolateral view [dotted line indicates the border of microtomentum] **11−12** *Centrophlebomyia anthropophaga* (Italy) **13−14** *Centrophlebomyia orientalis* (India) **15** *Centrophlebomyia anthropophaga* (Italy), detail of head in anterodorsal view [arrow indicates microtomentum on parafacial].

**Figures 16−21.**
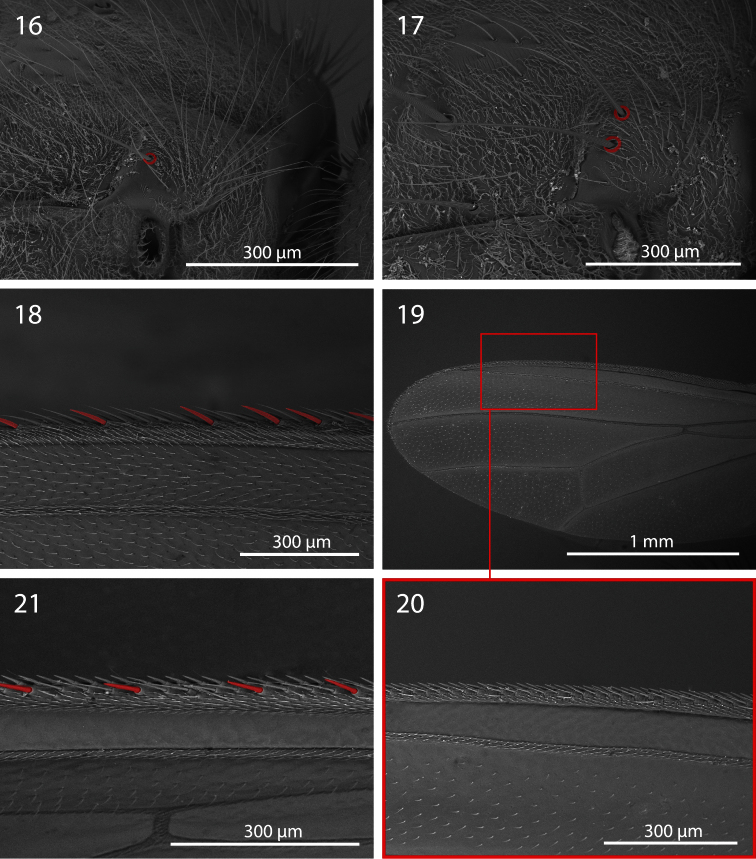
*Centrophlebomyia* spp. **16−17** *Centrophlebomyia anthropophaga* (Italy), anterior part of thorax in dorsolateral view [red circles indicate postpronotal setae] **16** male **17** female **18−21** Wing **18** *Centrophlebomyia anthropophaga* (Italy), detail of third costal sector (Cs_3_) [in red the costal spine-like setae] **19−20** *Centrophlebomyia grunini* (Russian Far East) **21** *Centrophlebomyia orientalis* (India), detail of third costal sector (Cs_3_).

**Figures 22−26.**
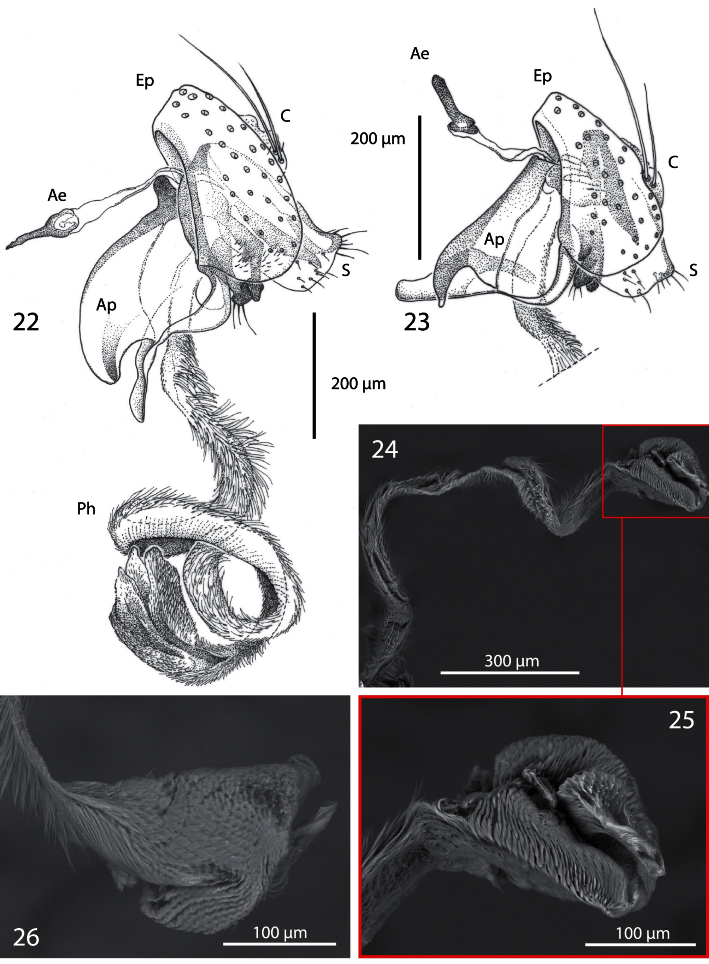
Male terminalia of *Centrophlebomyia* spp. [**Ae** = ejaculatory apodeme; **Ap** = phallapodeme; **C** = cerci; **Ep** = epandrium; **Ph** = phallus; **S** = surstylus] **22** *Centrophlebomyia anthropophaga* (Italy) **23** *Centrophlebomyia orientalis* (India) **24−26** *Centrophlebomyia anthropophaga* (Italy), phallus.

**Figures 27−29.**
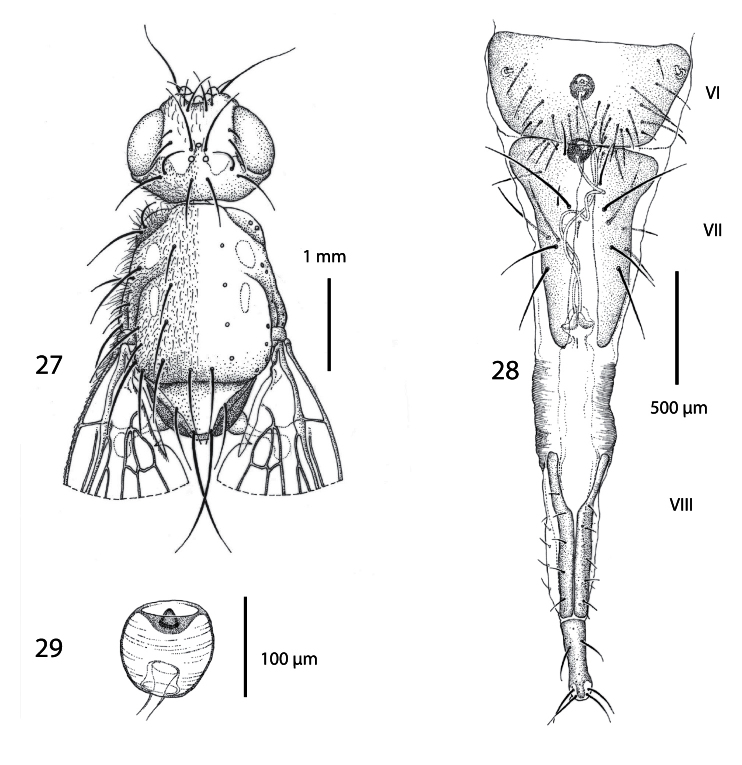
Female of *Centrophlebomyia anthropophaga* (Italy) **27** head and thorax in dorsal view **28** ovipositor in dorsal view **29** spermatheca.

**Figures 30−34.**
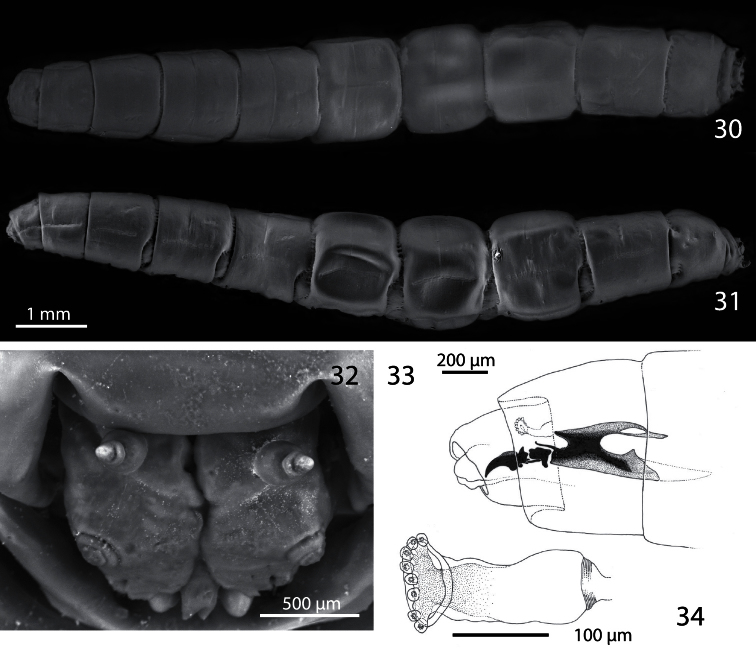
Third instar of *Centrophlebomyia anthropophaga* (Italy) **30** habitus in dorsal view **31** habitus in lateral view **32** head in frontal view **33** cephalopharyngeal skeleton in lateral view **34** aQnterior spiracle in lateral view.

**Figures 35−38.**
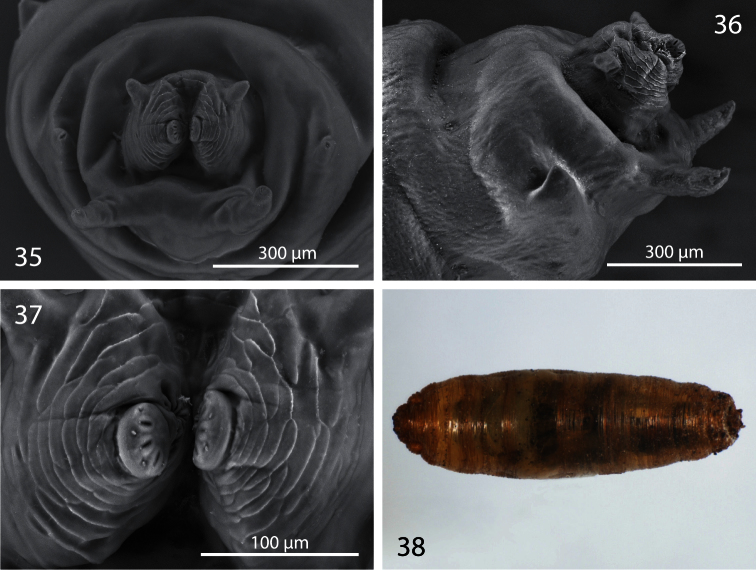
Third instar and puparium of *Centrophlebomyia anthropophaga* (Italy) **35** last segment bearing posterior spiracle in posterior view **36** last segment bearing posterior spiracle in lateral view **37** posterior spiracles in posterior view **38** puparium in dorsal view.

### Included species

#### Centrophlebomyia anthropophaga

(Robineau-Desvoidy, 1830)

http://species-id.net/wiki/Centrophlebomyia_anthropophaga

Figs 1−4 , 11, 12, 15 −18 , 22, 24 −38

Thyreophora anthropophaga Robineau-Desvoidy 1830: 623 – type locality: Paris (France).

##### Type material examined.

Neotype (designated below). Male (ZMUC), here designated, from Sardinia, Italy and bearing the following labels: SARDEGNA / Belvì (NU) / 10.X.’84 [39°57.889'N, 9°11.111'E] // Neotype ♂ / *Thyreophora* / *anthropophaga* / Robineau-Desvoidy, 1830 / M. Mei & P. Cerretti des. 2013 // *Centrophlebomyia* / *anthropophaga* / (Robineau-Desvoidy, 1830) / M. Mei & P. Cerretti det. 2013).

**Other material examined**. 5 ♂♂, 5 ♀♀, same data as neotype. 35 ♂♂, 3 ♀♀, Italy, Abruzzo, L’Aquila province, Riserva Naturale Orientata Monte Velino, Mandridi, 42°7.696'N, 13°22.247'E, 1270 m, 11.XI.2005, G. Lo Giudice, M. Mini, A. Vigna Taglianti legit. 20 ♂♂, 13 ♀♀, same data but 9.XII.2005; 13 ♂♂, 4 ♀♀, same data but 25.I.2006; 7 ♂♂, 1 ♀♀, same data but 25.X.2006; 2 ♂♂, same data but 9.XI.2006; 3 ♂♂, 5 ♀♀, same data but 16.XI.2006; 6 ♂♂, 4 ♀♀, same data but 30.XI.2006; 2♂♂, same data but XI.2006 reared from larvae, (see below); 13 ♂♂, 2 ♀♀, same data but 14.X.2007; 2 ♂♂, same data but 18.XI.2008.

##### References.

Michelsen 1983; Contini and Rivosecchi 1993; Martín-Vega et al. 2010.

##### Remarks.

Specimens from Sardinia were collected from a bag of dead, decaying snails (Contini and Rivosecchi 1993). Specimens from central Italy were collected with hand net (adults) and pitfall traps (adults, larvae) filled with a saturated solution of water and salt (NaCl), in a large fenced area where a feeding station (“vulture restaurant”) was kept for a population of griffon vultures living in the Nature Reserve. Twenty pitfall traps were placed around dead and dismembered sheep (front and rear quarters without skin and guts).

##### Distribution.

?France (Paris), Italy (Sardinia, Central Apennines).

##### Redescription.

**Colouration**. Head, including antenna and palpus, usually reddish yellow; sometimes dorsal half of occiput black. Occiput, ocellar triangle, genal dilation, and parafacial covered with microtomentum. When seen in dorsal view, the microtomentum that covers the occiput anteriorly outlines a three-pointed crown on the frons between the medial posterior margin of eyes (Figs 1, 3, 11, 12); middle tip of crown corresponds to anterior ocellus; lateral tips, laterally confined by eyes, end about level with posterior ocelli or slightly anteriorly. Prementum black. Postpronotum at least partly reddish laterally (reddish colour usually not visible in dorsal view). Scutum black in ground colour, covered with thin microtomentum except around base of dorsocentral setae and two lateral, longitudinal shiny vittae, widely interrupted at level of transverse suture (Figs 1, 27) (suture well developed laterally up to level of dorsocentral row). Scutellum at least apically yellow. Legs usually entirely yellow, rarely tarsi darkened. Abdomen usually entirely yellow or light brown, but can vary from dark brown to shiny black dorsally in some females. Setae of whole body black. Wing hyaline.

**Head** (Figs 1−4, 11−12, 15, 27). Head about as wide as thorax. Eye almost round. Frons 2.0−2.5 times as wide as an eye in dorsal view. Parafacial 1/2–2/3 as wide as first flagellomere, both measured at mid length. Gena, in profile, 0.33–0.65 times as high as eye. Medial vertical seta well developed, reclinate. Lateral vertical seta well developed, about 4/5 of medial vertical seta, lateroclinate. One or (usually) two upper reclinate orbital setae; when two are present, then anterior one at most 1/3 as long as posterior seta, and distinctly thinner. Postocellar seta strong and reclinate, subequal in size to ocellar and medial vertical setae. Ocellar seta proclinate. Anterior margin of frons with 2–3 pairs of pro- and medioclinate, strong frontal setae. Fronto-orbital plate with scattered, short, proclinate or medioclinate setae, between posteriormost upper reclinate orbital seta and distal margin of pedicel. Vibrissa double, very strong. Antenna shorter than height of facial ridge; first flagellomere 1.3–2.0 times as long as pedicel. Occiput and genal dilation covered with scattered black setae. Palpus well developed, apically clavate, covered with fine black setae.

**Thorax** (Figs 1, 3, 16, 17, 27). Thorax covered with fine black setulae, those on scutum distinctly shorter than those on pleurae. Postpronotum with or without 1–2 very fine setae in male (Fig. 16), usually with 2, relatively strong setae in female (Fig. 17). One strong presutural and 2 postsutural supra-alar setae; posterior postsutural supra-alar seta short and thin. One presutural and 3 postsutural dorsocentral setae (Figs 1, 27) (2 postsutural dorsocentral setae may occur in smaller sized male specimens (Fig. 3)). One, short and weak, prescutellar acrostichal seta. Scutellum dorsally flat to slightly concave (ground plan trait of the Thyreophorina, McAlpine, 1977), more or less elongated posteriorly, with one lateral seta and one apical seta (Figs 1, 3); lateral seta usually much smaller than apical seta. Shape and size of scutellum strongly variable (Figs 1, 3, 27) between sexes and between males of different sizes (Figs 1, 3). Two notopleural setae. One anepisternal seta. One katepisternal seta. Legs robust, covered with long and fine setulae. Mid tibia with 3–5 robust ventral preapical setae. Claws well developed in both sexes, about as long as fifth tarsal segment in male, varying in length between 0.5 and 0.7 times as long as fifth tarsal segment in female. Ventral row of costal setae (specifically CS_3_) characterized by the presence of some longer and stouter setae placed at more or less regular intervals (Fig. 18).

**Abdomen**. Male: more or less elongated; tergite 1 laterodorsally covered with short erect hair-like setae, medially bare; tergites 2 and 3 laterodorsally and ventrally covered with long, hair-like setae that become shorter toward the midline of tergites. Tergites 4 and 5 evenly covered with long, erect hair-like setae. Female: abdominal setae distinctly shorter.

**Male terminalia** (Figs 22, 24−26). Epandrium short and convex. Surstyli massive, almost touching each other posteromedially; distal margin of surstylus slightly bent posteriorly. Cerci very small, bearing long setae. Phallapodeme, in lateral view, very large with an evenly convex dorsal margin (Fig. 22). Pregonite well sclerotized, relatively narrow and slightly bent posteriorly; basally fused to hypandrium; pregonite with 1−2 fine setae distally. Postgonite very long, well sclerotized and evenly bent anteriorly. Pregonite and postgonite pincer-like in relative position, almost touching each other distally. Epiphallus attached basally and well developed. Basiphallus very long, tubular, covered with fine pubescence and membranous. Distiphallus massive, slightly sclerotized, covered with fine pubescence as in basiphallus; distiphallus with two large laterodistal lobes.

**Female terminalia** (Figs 28−29). Ovipositor long and telescopically retracted within fifth segment. Tergites 6 and 7 relatively wide and more or less flattened. Tergite 8 longitudinally divided into two halves. Cerci not differentiated. Two rounded and well sclerotized spermathecae.

##### Description of third instar and puparium.

Both the larva and puparium of *Centrophlebomyia anthropophaga* (Figs 30−38) correspond well to features given by McAlpine (1977), Ozerov (2000) and Ozerov and Norrbom (2010) for other piophilids and by Freidberg (1981) for *Centrophlebomyia furcata*. Here we provide additional information not given in previous descriptions. Nearly all the segments of the third instars have a lateral “dotted” line composed of microscopic, concave structures which may be sensory organs (Fig. 31). Their shape and position suggest that they may be mechanoreceptors of pressure or stretching. These structures have not been noted in previous descriptions of piophilid larvae; they were either overlooked or are unique to *Centrophlebomyia anthropophaga*.

##### Notes on larval development.

On April 5^th^ (n=15) and May 3^rd^ (n=7), 2006, several mature larvae were collected from the soil a few centimetres below the sheep quarters used as bait for the pitfall traps set in the “vulture restaurant” (see above under “Remarks”). The larvae were then transferred into two petri dishes (12 cm diameter): one filled with potting soil, the other with natural soil collected with the larvae from under the carcass. Moisture was provided each week until midsummer. All larvae remained active, though only slightly so, during this time. By June 1^st^, five out of 22 larvae had died. The loss of larvae continued steadily and by the beginning of September only six larvae were left, three in the potting soil and three in the natural soil. In early October 2006, two puparia were found in each dish and all the remaining larvae were dead. The four puparia and small amounts of soil were isolated in smaller dishes. An adult male emerged in November from one of the puparia in the natural soil, and another adult (possibly a male) was found dead in its puparium in the potting soil. The remaining two puparia failed to produce adults.

Our observations are consistent with those of Freidberg (1981; 2010 pers. comm.) on *Centrophlebomyia furcata* larvae reared in Israel. Mature *Centrophlebomyia furcata* larvae remained buried in the soil through spring and summer, estivating in this stage or as prepupae, and pupariated at the beginning of autumn. The larvae did not feed but were still more or less active. Most of the larvae died during the summer months and only very few adults emerged in October.

#### Centrophlebomyia furcata

(Fabricius, 1794)

http://species-id.net/wiki/Centrophlebomyia_furcata

Figs 5−6

Musca furcata Fabricius, 1794: 343 – type locality: “habitat in Gallia”.
Thyreolepida cinerea Sack, 1939: 4 – type locality: “Rehoboth [Rehovot] bei Jaffa” (Israel).

##### Material examined.

1 ♀, 1 ♂, Israel, Tel Aviv, 17.XII.1977, A. Freiberg legit (MZUR); several males and females, same data (TAU). 16 ♂♂, 9 ♀♀, Italy, Latium, Monti della Tolfa, Mount S. Ansino, 332 m, 42°03'51.85"N, 11°59'47.05"E, 28.XII.2011, M. Mei legit, on dead sheep (MZUR).

##### References.

Hendel 1903; McAlpine 1977; Freidberg 1981; Ozerov 2000; Martín-Vega et al. 2010.

##### Distribution.

Europe: Austria, Cyprus, France, Germany, Greece, Italy, Spain, United Kingdom; North Africa: Algeria; Middle East: Turkey, Israel.

#### Centrophlebomyia grunini

(Ozerov, 1984) comb. n.

http://species-id.net/wiki/Centrophlebomyia_grunini

Figs 7−8 , 19−20

Protothyreophora grunini Ozerov 1984a: 466 – type locality: “Aмурская област, Г. ЗеЯ” [= Amur region, near Zeya].

##### Material examined.

1 ♂,1 ♀, each bearing the following labels: [Russia] Aмурская обл[аст] / Г. ЗеЯ [= Amur region, Zeya.] 1.VIII.1981 / A. Ozerov / PARATYPE (ZMUC).

##### References.

Ozerov 1984a, 2000.

##### Distribution.

Russian Far East.

#### Centrophlebomyia orientalis

(Hendel, 1907) stat. r.

Figs 9−10 , 13−14 , 21 , 23

Centrophlebomyia orientalis Hendel 1907: 243 – type locality: “Indien, Darjeeling, am Himalaya”.
Centrophlebomyia orientalis Treated as junior synonym of *anthropophaga*Robineau-Desvoidy, 1830 by Martín-Vega et al. (2010: 611).

##### Type material examined.

Holotype male, bearing the following labels: [INDIA] Darjeeling / Juni / Frusthofer leg. // *Centrophlebomyia* / *orientalis* / Hendel [handwritten] // Type [red label] // Holotypus [red label] // *Pseudotyreo* - / *phora orientalis* / Hendel / A. Ozerov det., 1984 (NHMW).

##### Other material examined.

INDIA (Kashmir) Gulmarg, 2600–3000 m, 17.VIII 5.IX.1978: 2 ♂♂, 1 ♀ (ZMUC).

##### References.

Hendel 1907; Michelsen 1983; Ozerov 2000.

##### Distribution.

Northern India (Darjeeling, Kashmir).

### Key to species of *Centrophlebomyia*

**Table:** 

1	Scutum mostly shiny black with two large, longitudinal, silver stripes of microtomentum aligned with, and along the entire length of, dorsocentral setae (Fig. 7 [where the three shiny black vittae are yellow highlighted]). Head almost entirely smooth, katepisternum without microtomentum. Costal vein without spine-like setae (Figs 19−20). [Gena 0.44–0.47 times as high as eye in lateral view (Fig. 8)]	*Centrophlebomyia grunini* (Ozerov) comb. n.
–	Scutum usually more extensively covered with microtomentum, and not showing the pattern described above (Figs 1, 3, 5, 9, 27). Head microtomentose at least on medial dorsal portion of postcranium, katepisternum at least partly microtomentose (e.g., dorsal and posterior to base of katepisternal seta). Ventral row of costal setae characterized by some longer and stouter (spine-like) setae placed at more or less regular intervals, especially on Cs_3_ (Figs 18, 21)	2
2	Gena 0.68–0.95 times as high as eye in lateral view (Fig. 6). One upper reclinate orbital seta (Figs 5, 6). Genal dilation and occiput entirely covered with microtometum. Occipital microtomentum extends anteriorly on fronto-orbital plate as shown in Fig. 5 [in red]. One presutural dorsocentral seta (Fig. 5). Katepisternum entirely covered with microtomentum. Male: postpronotal setae not differentiated (Fig. 5)	*Centrophlebomyia furcata* (Fabricius)
–	Gena 0.33–0.65 times as high as eye in lateral view (Figs 2, 4, 10). Two upper reclinate orbital setae (exceptionally only one in *Centrophlebomyia anthropophaga*) (Figs 2, 4, 10). Occipital microtomentum never extending anteriorly on fronto-orbital plate (Figs 1, 3, 9 [in red]). Occipital microtomentum extends anteriorly on frons as shown in Figs 1, 3, 9, 11−14 [in red]. One or two presutural dorsocentral setae. Katepisternum varying from entirely covered with microtomentum to largely smooth. Male: postpronotal setae well developed	3
3	Genal dilation and occiput entirely covered with microtometum (Figs 1, 3, 11, 12 [in red]). Occipital microtomentum extends anteriorly on frons as shown in Figs 1, 3, 11, 12 [in red]. One presutural dorsocentral seta (Figs 1, 3, 27). Katepisternum entirely covered with thick microtomentum. Usually two upper reclinate orbital setae, the anterior one very short and weak (Figs 2, 4) (only one upper reclinate orbital seta present in a few specimens from Sardinia). Male: phallapodeme, in lateral view, with an evenly convex dorsal margin and posterior tip lobe-like (Fig. 22); one or rarely two hair-like postpronotal setae	*anthropophaga* (Robineau-Desvoidy)
–	Genal dilation entirely smooth, occiput largely without microtomentum laterally (Figs 9, 13, 14 [where microtomentum is red highlighted]). Occipital microtomentum extends anteriorly on frons as shown in Figs 9, 13, 14 [in red]. Two presutural dorsocentral setae (Fig. 9). Katepisternum almost entirely smooth. Two upper reclinate orbital setae, the anterior one distinctly shorter but somewhat stout (Fig. 10). Male: phallapodeme, in lateral view, with an almost straight dorsal margin, posterior tip narrow and hook-like (Fig. 23); two strong postpronotal setae	*orientalis* (Hendel)

## Discussion and conclusion

### The neotype designation of “*anthropophaga*”

Martín-Vega et al. (2010) recognised two species in the genus *Centrophlebomyia*: *Centrophlebomyia furcata* (Fabricius, 1794), found in Europe, North Africa and the Near East, and *Centrophlebomyia anthropophaga* (Robineau-Desvoidy, 1830) (with *Centrophlebomyia orientalis* Hendel, 1907 in synonymy), known from Paris (France), Sardinia (Italy) and northern India.

*Centrophlebomyia anthropophaga* was described by Robineau-Desvoidy (1830, in *Thyreophora*) “based solely on his memory of specimens he had observed in large numbers destroying preparations of human muscles, ligaments and bones in the Paris School of Medicine in August 1821” (Martín-Vega et al. 2010: 611). The original description is too general and inadequate to properly characterize the species and, as usual for Robineau-Desvoidy, there are no illustrations. No type material exists because no specimens were collected at the time the observations were made. Hence, the true identity of this nominal species has remained uncertain. It was treated as an “invalid” (in the sense of “unavailable”) name in the Catalogue of Palaearctic Diptera (Papp, 1984), but was correctly interpreted as an available name according to the present *Code* (ICZN 1999) by Martín-Vega et al. (2010). Just prior to the appearance of the Catalogue, the name *anthropophaga* was resurrected by Michelsen (1983) (“with a rather bold assumption”, see Martín-Vega et al. 2010: 611) for specimens of a thyreophorine species collected in Kashmir. This name was used also by Contini and Rivosecchi (1993) for specimens of *Centrophlebomyia* collected in Sardinia that were considered conspecific with those of Michelsen (see also Rivosecchi 2008). The existence of *Centrophlebomyia orientalis*, a nominal species briefly described by Hendel (1907) from Darjeeling (West Bengal, India), appears to have escaped the attention of these authors also because not listed in the catalogues. Ozerov (2000), in his review of the Palaearctic Piophilidae, treated the specimens from Kashmir (Michelsen 1983) and Sardinia (Contini and Rivosecchi 1993) as *Centrophlebomyia orientalis*. Ozerov (2004) later recognized only *Centrophlebomyia furcata* in Europe and recorded it from Sardinia and Czech Republic. In the most recent treatment of *Centrophlebomyia*, Martín-Vega et al. (2010) considered *Centrophlebomyia* from Sardinia and India as conspecific under the name *Centrophlebomyia anthropophaga* and formally placed *Centrophlebomyia orientalis* in synonymy. It is noteworthy that Contini and Rivosecchi (1993) never studied the material of Michelsen (1983), only his drawings and descriptions, and neither Ozerov (2000) nor Martín-Vega et al. (2010) studied the Sardinian specimens.

Our study shows that specimens of *Centrophlebomyia* from Sardinia and central Italy are conspecific and are different from both *Centrophlebomyia orientalis* and *Centrophlebomyia furcata*, so we here propose to remove *orientalis* Hendel from synonymy with *anthropophaga* Robineau-Desvoidy.

Furthermore, considering the confused taxonomic and nomenclatural situation described above, created by Michelsen’s (1983) resurrection of the name *anthropophaga* and by the subsequent repeated use of this name (Contini and Rivosecchi 1993; Martín-Vega et al. 2010), we see a need to designate a neotype to fix once and for all the identity of this nominal species.

Considering that:

i) *Centrophlebomyia anthropophaga*’s original description is vague and insufficient, and no type specimens ever existed in collections as no actual specimen was ever collected;

ii) The name *anthropophaga* is nomenclaturally available according to the *International Code of Zoological Nomenclature* (1999);

iii) Michelsen (1983) convincingly showed that Robineau-Desvoidy’s original description of *Centrophlebomyia anthropophaga* is a poor match with *Centrophlebomyia furcata* (the only other european species known at the time) but provides a reasonable fit with his Kashmir material (i.e., with *Centrophlebomyia orientalis*);

iv) Robineau-Desvoidy’s original description of *Centrophlebomyia anthropophaga* is not inconsistent also with characters shown by the Italian specimens;

v) The *Centrophlebomyia* from Italy is not conspecific with *Centrophlebomyia orientalis*, the species dealt with by Michelsen (1983), nor with *Centrophlebomyia furcata*, and is actually an unnamed species;

vi) The specimens from Sardinia were already referred to under the name *anthropophaga* by Contini and Rivosecchi (1993) and Martín-Vega et al. (2010);

we select as neotype a male specimen from Sardinia chosen among those studied by Contini and Rivosecchi (1993). As no French or European material of *Centrophlebomyia anthropophaga* (*sensu* this paper) is known to exist, our choice of a Sardinian specimen is essentially as near as practicable to the original type locality, thereby fulfilling Article 75.3.6 of the *Code* (ICZN 1999). We refrain from proposing a new name for the Italian species because it would not resolve the problem of the name *anthropophaga*, that would remain available and valid, though not associated with type specimens nor unambiguously attributable to a known species.

### *Protothyreophora* as a junior synonym of *Centrophlebomyia*

The thyreophorine genus *Protothyreophora* was proposed by Ozerov (1984a) for the single species *Protothyreophora grunini*, stressing that it is distinct from *Centrophlebomyia* in having two upper reclinate orbital setae instead of only one, two presutural dorsocentral setae instead of one, a distinctive pattern of thoracic microtomentum and the costal vein without any spine-like setae distally. This shows that Ozerov based his definition of *Centrophlebomyia* exclusively on characters of the type species *Centrophlebomyia furcata*. Our examination of specimens of *Centrophlebomyia orientalis* shows that this species has 2 upper reclinate orbital setae and 2 presutural dorsocentral setae, as in *Centrophlebomyia grunini*. Moreover, the spine-like setae on the costa in thyreophorines may vary also between males and females of the same species (*Centrophlebomyia furcata*), and be present or absent in different species of the same genus (*Piophilosoma* Hendel). The characteristic pattern on the thorax of *Centrophlebomyia grunini* (Fig. 7), with two longitudinal bands of grey microtomentum along the line of the dorsocentral setae, contrasting with the remaining smooth surface, can be interpreted as having evolved from the pattern found in other species of *Centrophlebomyia* (small smooth areas lacking microtomentum, feebly developed in *Centrophlebomyia furcata* and *Centrophlebomyia anthropophaga* but much more extensive in *Centrophlebomyia orientalis*) (Figs 1, 5, 9).

For these reasons, we consider the monotypic genus *Protothyreophora* as a junior synonym of *Centrophlebomyia*.

### Concluding remarks

The European thyreophorine species have always been considered very rare insects and have famously also been considered as locally or even globally extinct (Cogan and Dear 1975; Fontaine et al. 2007; Pape 2009). We still know almost nothing about the biology, ecology and distribution of these flies, due both to their apparent rarity and the lack of targeted research. It was known for a long time (e.g., Robineau-Desvoidy 1842, 1849) that thyreophorine species have a winter phenology and are preferentially associated with large carcasses in an advanced state of decay, but for many decades there was no attempt to use this information to launch determined searches for these insects. The first detailed study was that of Freidberg (1981) on the biology of *Centrophlebomyia furcata* in Israel. Additional data were published on *Centrophlebomyia grunini* in the Russian Far East by Ozerov (1984b), and only in the last few years are we beginning to understand the ecology of *Thyreophora cynophila* after its rediscovery in Spain (Carles-Tolrá et al. 2010; Martín-Vega and Baz 2011). Further studies are necessary to increase our knowledge of the ecology of these flies and to assess their conservation status.

It would seem that these flies may also be relatively abundant in sites where they are present (Freidberg 1981; Carles-Tolrá et al. 2010; and authors pers. obs.), but in general they seem to be very localized and living at low population densities. Martín-Vega and Baz (2011) noted the role of “vulture restaurants” and protected areas in maintaining populations of bone-skippers, but they considered other measures to be necessary as well, such as different management practices of livestock and wild ungulates aimed at naturally generating a constant availability of large carcasses. As mentioned above, *Centrophlebomyia anthropophaga* was also collected by us in a “vulture restaurant”, where it was present in abundance, but we have no further information on the presence of this species elsewhere in central Italy. *Centrophlebomyia furcata* was collected by one of us (MM) in a rather remote area characterized by a considerable and continuous presence of wild cattle, sheep and horses, and where carcasses of such large animals that died naturally are regularly present.

It would also be interesting to assess the role of food sources other than large mammal carcasses in maintaining natural populations of these flies. Other studies have shown that thyreophorine species have also been collected from a bag of dead decaying snails (*Centrophlebomyia anthropophaga*: Contini and Rivosecchi 1993), dead rodents (*Centrophlebomyia grunini*: Ozerov 1984a), traps baited with dead squids (*Thyreophora cynophila*: Martín-Vega et al. 2010), and a dead bird (Carles-Tolrá 2011). This would seem to indicate that these alternative food sources may play some role in maintaining populations of these insects.

We hope that the interest generated by the recent rediscovery of these flies will result in further studies on their reproductive biology and ethology, as combining the results of such studies with the already available morphological data may help explain the great polymorphism observed in adult males.

## Supplementary Material

XML Treatment for Centrophlebomyia

XML Treatment for Centrophlebomyia anthropophaga

XML Treatment for Centrophlebomyia furcata

XML Treatment for Centrophlebomyia grunini

XML Treatment for Centrophlebomyia orientalis

## Appendix 1

Online media coverage of recent findings of Thyreophorina in Europe:

http://www.youtube.com/watch?v=gwqL02RstAw

http://www.youtube.com/watch?v=QW-tOF0WtXA

http://www.youtube.com/watch?v=zXgIrfwwRvg

http://www.youtube.com/watch?v=Bmqgf-TaJsQ

http://www.youtube.com/watch?v=eCm9I__G7HI

http://www.youtube.com/watch?v=p1QcpliZm3g

http://www.youtube.com/watch?v=gGqGAEUDFUU

http://www.youtube.com/watch?v=NnECVsursMs

http://www.diptera.info/forum/viewthread.php?forum_id=5&thread_id=46137

http://www.diptera.info/forum/viewthread.php?forum_id=5&thread_id=26429

http://www.diptera.info/forum/viewthread.php?forum_id=22&thread_id=12704

http://www.diptera.info/forum/viewthread.php?forum_id=5&thread_id=11348

http://www.diptera.info/forum/viewthread.php?forum_id=5&thread_id=32315

http://www.diptera.info/forum/viewthread.php?forum_id=12&thread_id=32317

http://www.diptera.info/forum/viewthread.php?forum_id=5&thread_id=26429

http://news.bbc.co.uk/earth/hi/earth_news/newsid_9008000/9008585.stm

## References

[B1] Carles-Tolrá M (2011) Primera cita de *Thyreophora cynophila* (Panzer) sobre cadáveres de aves (Diptera: Piophilidae: Thyreophorina). Boletín de la Sociedad Entomologica Aragonesa, 49: 355−356.

[B2] Carles-Tolrá M Cañete Saiz FJ (2012) Primera cita de *Thyreophora cynophila* (Panzer) para la provincia de Cuenca (España) (Diptera: Piophilidae: Thyreophorina). Boletín de la Sociedad Entomologica Aragonesa, 50: 254.

[B3] Carles-Tolrá M Compaired F Vlasco J (2011) *Thyreophora cynophila* (Panzer), *Centrophlebomyia furcata* (fabricius) and other dipterans associated to winter carcasses (Insecta: Diptera). Boletín de la Sociedad Entomologica Aragonesa, 48: 217−220.

[B4] Carles-Tolrá M Rodriguez PC Verdù J (2010) *Thyreophora cynophila* (Panzer, 1794): collected in Spain 160 years after it was thought to be extinct (Diptera: Piophilidae: Thyreophorini). Boletín de la Sociedad Entomologica Aragonesa, 46: 1−7.

[B5] Cerretti P (2010) I tachinidi della fauna italiana (Diptera Tachinidae), con chiave interattiva dei generi ovest-paleartici. Volume I, 573 pp., Volume II, 339 pp. + CD-ROM. Cierre Edizioni, Verona.

[B6] Cerretti P Pape T (2012) Phylogenetics and taxonomy of *Ventrops*— the largest genus of Afrotropical Rhinophoridae (Diptera). Invertebrate Systematics, 26(3): 274−292. doi: 10.1071/IS12001

[B7] Cogan BH Dear JP (1975) Additions and corrections to the list of British Acalypterate Diptera. Entomologist’s Monthly Magazine, 110: 173−180.

[B8] Contini C Rivosecchi L (1993) Sulla presenza in Sardegna di *Centrophlebomyia anthropophaga* (Rob. Desv., 1830) (*sensu* Michelsen, 1983) (Diptera Thyreophoridae). Fragmenta entomologica, 25: 275−280.

[B9] Fabricius JC (1794) Entomologia Systematica emendata et aucta. Vol. 4. Hafniae [= Copenhagen], 472 + i-vi + 1 pp.

[B10] Fontaine B Bouchet P van Achterberg K (2007) The European union’s 2010 target: putting rare species in focus. Biological Conservation, 139: 167−185. doi: 10.1016/j.biocon.2007.06.012

[B11] Freidberg A (1981) Taxonomy, natural history and immature stages of the bone-skipper, *Centrophlebomyia furcata* (Fabricius) (Diptera: Piophilidae, Thyreophorina). Entomologica Scandinavica, 12: 320−326. doi: 10.1163/187631281794709728

[B12] Gomez-Gomez A Diaz-Aranda LM Michelsen V (2008) Rediscovery of *Centrophlebomyia furcata* (Fabricius, 1794) (Diptera: Piophilidae) in Europe. Studia dipterologica, 15(1/2): 231–237.

[B13] Hendel F (1903) *Centrophlebomyia* nov. gen. Thyreophorinae. Zeitschrift für systematische Hymenopterologie und Dipterologie, 3: 215−216.

[B14] Hendel F (1907) Neue und interessante Dipteren aus der kaiserl. Museum in Wien. Wiener Entomologische Zeitung 26(7–9): 245.

[B15] International Commission on Zoological Nomenclature (1999) International Code of Zoological Nomenclature. Fourth edition adopted by the International Union of Biological Sciences. International Trust for Zoological Nomenclature, London. xxix + 306 pp.

[B16] Lo Giudice G (2007) Studio di una comunità di Ditteri necrofagi nel Massiccio del Velino-Sirente (Appennino Centrale). Degree thesis (not published), supervisor A. Vigna Taglianti, Facoltà di Scienze Matematiche, Fisiche e Naturali, Università degli Studi di Roma “La Sapienza”, 114 pp.

[B17] Martín-Vega D Baz A Michelsen V (2010) Back from the dead: *Thyreophora cynophila* (Panzer, 1798) (Diptera: Piophilidae) “globally extinct” fugitive in Spain. Systematic Entomology, 35 (4): 607-613. doi: 10.1111/j.1365-3113.2010.00541.x

[B18] Martín-Vega D Baz A (2011) Could the “vulture restaurants” be a lifeboat for the recently rediscovered bone-skippers (Diptera, Piophilidae). Journal of Insect Conservation, 15(5): 747–753. doi: 10.1007/s10841-011-9429-0

[B19] McAlpine JF (1977) A revised classification of the Piophilidae, including “Neottiophilidae” and “Thyreophoridae” (Diptera: Schizophora). Memoirs of the Entomological Society of Canada, 103: i–vi, 1–66.

[B20] Merz B Haenni JP (2000) 1. 1. Morphology and terminology of adult Diptera (other than terminalia). In: Papp L, Darvas B (Eds) Contribution to a Manual of Palaearctic Diptera (with special reference to flies of economic importance). Vol. 1. General and Applied Dipterology. Science Herald, Budapest: 21−51.

[B21] Michelsen V (1983) *Thyreophora anthropophaga* Robineau-Desvoidy, an “extinct” bone-skipper rediscovered in Kashmir (Diptera: Piophilidae, Thyreophorina). Entomologica Scandinavica, 14: 411-414. doi: 10.1163/187631283X00173

[B22] Ozerov AL (1984a) A new Palearctic genus of the family Thyreophoridae (Diptera) from the Soviet Far East [in Russian]. Zoologicheskii Zhurnal, 63(3): 464–466.

[B23] Ozerov AL (1984b) К биологии *Protothyreophora grunini* Ozerov (Diptera, Thyreophoridae). Доклады высшей школы. Биологические науки. № 4, С. 39–41. [The biology of *Protothyreophora grunini* Ozerov (Diptera, Thyreophoridae). Reports of the High School. Biological sciences, 4: 39–41.]

[B24] Ozerov AL (2000) A.9. Family Piophilidae. In: Papp L Darvas B (Eds). Contributions to a manual of Palearctic Diptera. Appendix. Science Herald, Budapest: 355-365.

[B25] Ozerov AL (2004) Fauna Europaea: Piophilidae. In: Pape T (Ed) Fauna Europaea: Diptera Brachycera. Fauna Europaea version 2.4, http://www.faunaeur.org [accessed 23 January 2013]

[B26] Ozerov AL Norrbom AL (2010) Piophilidae. In: Brown BV, Borkent A, Cumming JM, Wood DM, Woodley NE, Zumbado M (Eds). Manual of Central American Diptera. Ottawa, Canada: National Research Council Press: 865−869.

[B27] Pape T (2009) Chapter 5. Palaearctic Diptera – from tundra to desert. In: Pape T, Bickel D, Meier R (Eds) Diptera Diversity: Status, Challenges and Tools. Brill, Leiden – Boston: 121−154. doi: 10.1163/ej.9789004148970.I-459.27

[B28] Papp L (1984) Family Thyreophoridae. Vol. 9. In: Soós Á, Papp L (Eds) Catalogue of Palearctic Diptera. Micropezidae-Agromyzidae. Akadémiai Kiadó, Budapest: 241−242.

[B29] Rivosecchi L (2008) Aggiunte e correzioni alle checklist di alcune famiglie di Ditteri della fauna italiana (Diptera). Bollettino della Società entomologica italiana 140 (2): 95-103

[B30] Robineau-Desvoidy JB (1830) Essai sur les myodaires. Mémoires présentés par divers Savans à l’Académie Royal des Sciences de l’Institut de France (Sciences Mathématiques et Physiques), (2) 2: 813 pp.

[B31] Robineau-Desvoidy JB (1842) Note sur le *Thyreophora cynophila*. Annales de la Société Entomologique de France 10, (1841): 273.

[B32] Robineau-Desvoidy JB (1849) Communication (Séance du 10 Janvier 1849). Annales de la Société Entomologique de France 7: iv−vi.

[B33] Sack P (1939) 62b. Thyreophoridae. In: Lindner E (Ed) Die Fliegen der palearktischen Region. Stuttgart: 1−7.

[B34] Stuckenberg BR (1999) Antennal evolution in the Brachycera (Diptera) with a reassessment of terminology relating to the flagellum. Studia dipterologica 6: 33−48.

[B35] Zaldivar Ezquerro C Rodrìguez PC Gòmez Vargas J (2011) *Thyreophora cynophila* (Panzer, 1798) (Diptera: Piophilidae: Thyreophorini): distribution area in La Rioja (Spain). Boletín de la Sociedad Entomologica Aragonesa 48: 403-405.

